# Antimicrobial stewardship program in a Malaysian district hospital: First year experience

**DOI:** 10.12669/pjms.324.9855

**Published:** 2016

**Authors:** Diana Yap Fui Sing, Yang Liang Boo, Roshalina Mukhlis, Pek Woon Chin, Fan Kee Hoo

**Affiliations:** 1 Diana Yap Fui Sing, Master of Clinical Pharmacy (UKM). Hospital Enche’ Besar Hajjah Khalsom, Jalan Kota Tinggi, 86000 Kluang, Johor, Malaysia; 2 Dr. Yang Liang Boo, MRCP (UK). Hospital Enche’ Besar Hajjah Khalsom, Jalan Kota Tinggi, 86000 Kluang, Johor, Malaysia; 3 Roshalina Mukhlis, Bachelor of Pharmacy (Hons) (UKM). Hospital Enche’ Besar Hajjah Khalsom, Jalan Kota Tinggi, 86000 Kluang, Johor, Malaysia; 4 Dr. Pek Woon Chin, MRCP (UK). Hospital Enche’ Besar Hajjah Khalsom, Jalan Kota Tinggi, 86000 Kluang, Johor, Malaysia; 5 Dr. Fan Kee Hoo, MRCP (UK). Faculty of Medicine and Health Sciences, Universiti Putra Malaysia, Serdang, Malaysia

**Keywords:** Antimicrobial stewardship, Anti-Infective agents, Drug resistance, Malaysia, Rural hospital

## Abstract

**Backgrounds & Objective:**

Antimicrobial resistance is an alarming public health threat that requires urgent global solution. Implementation of antimicrobial stewardship program (ASP) is an essential practice element for healthcare institutions in gate-keeping judicious antimicrobial use. This study highlighted the development, first year experience, and result of the implementation of ASP utilizing persuasive and restrictive approaches in a Malaysian district hospital.

**Methods:**

An observational study was conducted between January 2015 to December 2015 on implementation of ASP among hospitalized inpatients age 12 years old and above.

**Results:**

Recommendations were provided for 60% of cases (110 patients) with the average acceptance rate of 83.33%. Majority of the interventions were to stop the antimicrobial therapy (30.3%), and the most common audited antimicrobials was Piperacillin/Tazobactam (25.5%), followed by Meropenem (11.82%), Amoxicillin/Clavulanate and Vancomycin (8.18%) respectively. The concordance rate towards authorization policy was increased in 2015 (71.59% of cases) as compared before the implementation of ASP in 2014 (60.6% of cases). Restrictive enforcement under ASP had been shown to improve significantly adherence rate towards antimicrobials authorization policy (p-value: 0.004).

**Conclusion:**

ASP was successfully implemented in a district hospital. Future studies on its clinical outcomes are important to evaluate its effectiveness as well as focus on the improvement to the pre-existing strategies and measures.

## INTRODUCTION

Antimicrobial resistance is an alarming public health threat that requires urgent global solution.^1^ World Health Organization (WHO) had launched the first World Antibiotic Awareness Week in 2015, aimed to increase awareness of global antibiotic resistance and to encourage best practices in utilizing antimicrobials.^2^ Thus, the implementation of antimicrobial stewardship program (ASP) is an essential practice element for healthcare institutions in gate-keeping judicious antimicrobial use.^3^ It is a system involving a multidisciplinary team adapting evidence-based data and guidelines to formulate an overarching action plan to influence and guide rational antimicrobial prescribing.^4^

The emergence of antimicrobial resistance can render the first line antimicrobials to be ineffective, leading to the use of second or third line agents which may be more toxic and costly.^5^ In Malaysia, a significant 16% of increment in annual antimicrobial consumption was reported from 2009 to 2010.^5^ Furthermore, systemic use of antibacterial remained the highest ranked therapeutic group in our healthcare expenditure.^5^ Implementation of ASP is warranted to cope with irrational antimicrobial use, and thus, reducing antimicrobial expenditure and to curb the issue of antimicrobial resistance.^1^,^6^

Despite the overwhelming evidences that support the ASP in countries around the world,^4^,^7^,^8^ only a few hospitals in Malaysia had implemented a comprehensive ASP. Although ASP Protocol outlined by our Ministry of Health has defined the core elements to be included in ASP^5^, systematic approach and practical aspect for its implementation has not been described adequately. Moreover, to date, data pertinent to the outcome of ASP in Malaysia is scarce. A thorough literature search showed there was no published report on the implementation of ASP in a district hospital in Malaysia. This article highlights the practical aspect of the development, first year experience, and with special commentary on the result of process metrics post-implementation of ASP utilizing persuasive and restrictive approaches in a Malaysian district hospital.

## METHODS

### Setting

An ASP was implemented at a non-profit, government-funded, district hospital, equipped with 268 beds including intensive care, and consisted of 7 major disciplines (medical, surgical, orthopedic, pediatric, anesthetic, obstetric & gynecology, and emergency department). Before the initiation of ASP, routine prospective review on antimicrobial use was not carried out. In addition, the hospital did not have a resident infectious disease (ID) physician nor ID-trained pharmacist to support such monitoring.

After a baseline review of the hospital existing resources, procedures, and policies on antimicrobial use, a structured process for ASP had been developed locally at the end of year 2014. According to protocol outlined by our Ministry of Health, successful ASP contained a range of core strategies.^5^ This included leadership and accountability; formulation of antimicrobial stewardship (AMS) round; antimicrobial formulary restriction; audit and feedback as well as reporting and education.^5^ However, there was no ‘one-size-fits-all’ approach. Hence, the standardized ASP recommended by the guidelines may not necessarily be the best fit for any one of the institution. The structure of the ASP should therefore conform and adaptable to available facility resources. Utilizing it as the backbone, the unique and hospital specific program was initiated locally with the appointment and identification of the roles of each team member involved. In contrary to the recommendation by Infectious Disease Society of America (IDSA) ASP Guidelines that suggested that ASP be administered by either ID specialists or ID pharmacist^3^, the most daunting challenge in building of the AMS team in our hospital was none of these in-house personnel were available to oversee the program in our hospital. Hence, empowering an alternate physician with interest in infectious disease was vital to direct the team. Our AMS team comprised of a medical specialist who had been delegated as AMS director to lead the AMS team, other specialists, residents from various disciplines, dedicated pharmacists, clinical microbiologist, and infection control linked nurses. The AMS team was placed under a consultant physician who was assigned as the chairperson for the Hospital Infection and Antibiotic Control Committee (HIACC).

**Fig.1:**
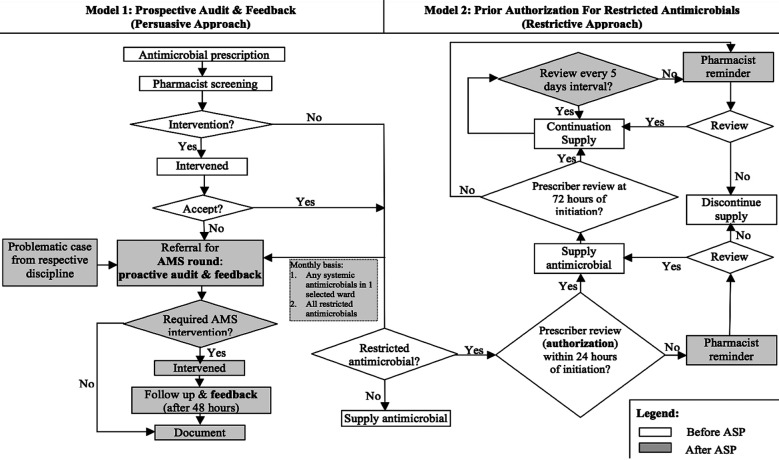
AMS workflow before and after ASP implementation.

By integrating the key activities recommended by the Malaysian ASP Protocol into existing hospital point of care, the characteristics of the local ASP program: institutional ASP workflow (Figure 1) tailored to our hospital setting, manual activity documentation (AMS referral form, clerking sheet, records), antimicrobials to be included under ASP restricted authorization policy (after taking into consideration of host factors and local resistance pattern) were defined and discussed among the team members and established in January 2015. Two proactive core strategies were adopted in our ASP: persuasive approach (prospective audit with intervention and feedback) and restrictive approach (enforcement on prior authorization for restricted antimicrobials). Based on the hospital’s capacity and human resources, the AMS round was conducted on monthly basis in addition to referral from respective disciplines during interval. Patients were recruited if they were started on restricted systemic antimicrobials, in which the list was expanded and modified with times (azithromycin, cefepime, cefoperazone/sulbactam, clindamycin, ertapenem, imipenem/cilastatin, meropenam, piperacillin/tazobactam, polymyxin E, vancomycin, cefotaxime, ceftazidime, cefoperazone, ciprofloxacin, and antifungals). Pediatric patients were excluded from review in this study. As the program evolved, all patients aged 12 years old and above, who were on any type of systemic antimicrobials, from a single monthly selected ward were included in the AMS round for review. This was carried out to ensure proper and rational use of common antimicrobials in our hospital besides restricted antimicrobials.

### Program Implementation

Four pharmacists were assigned to participate in this program. During the study period, these dedicated pharmacists would screen the antimicrobial prescriptions, prepare cases for monthly AMS review, accept AMS referrals from the clinicians and perform pharmacist’s intervention when appropriate. Furthermore, they also would coordinate mini AMS round apart from the monthly schedule upon referral in an as needed basis. A medical specialist had been assigned as the AMS director to lead the AMS round together with specialists from the other disciplines. Each round comprised of a focus review of clinical notes, investigation results, patients’ clinical progressions, the indication of the antimicrobial prescriptions, planned treatment duration, choice of antimicrobial, correct dosage, and appropriateness of antimicrobial therapy. Recommendations were communicated, discussed, and documented in patients’ files as medical record to the prescribing physician. An ID physician at a remote tertiary institution served as an off-site consultant to the AMS program when additional stewardship advices needed. The prescribing clinicians held the final decision regarding patient’s management.

Follow-up was carried out by AMS pharmacists at 48 hours’ post-intervention with prescribing clinicians and through patient’s chart review. It was considered accepted if they were enacted within 48 hours after the suggestions had been made.^9^,^10^ AMS recommendations which served as persuasive AMS approach were based on standardized, evidence-based practices as defined by national guidelines, other subspecialty practices as well as personal professional experiences. In this study, the recommendation was considered rejected if they were executed after this time frame. In addition, microbiology data focused on positive culture for resistant organisms were routinely reported for the attention of AMS team members for further review.

### Antimicrobial Prescribing Restriction

Limiting the unnecessary broad-spectrum antibiotic exposure is a practical and logical strategy in combating the emergence of resistance. The use of authorization policies in controlling the usage of restricted antimicrobial was implemented previously. However, low adherence rate had been observed. Thus, amendments on existing workflow (Fig.1) and educational reinforcement on the restricted antimicrobial policy were carried out throughout the study period to improve compliance. Furthermore, antimicrobials were generally initiated empirically before a patient’s full clinical picture was obtained. However, it was noted that prescribers tend not to revisit the antimicrobial selection after clinical and culture data became available. An antimicrobial ‘time-out’ policy hence provided an opportunity to evaluate and reassess the use of antimicrobial if it was still warranted or if it was still effective against identified organism. The prescribing clinicians were required to fill in the standardized manual controlled antimicrobial initiation forms that were completed with patients’ clinical details to justify the use for all restricted antimicrobials. Furthermore, they were also required to review the usage after 72 hours and at every five days intervals. An automatic antimicrobial ‘time-out’ with pharmacists’ reminder would be executed at 24-hours and at 72-hours post-initiation of restricted antimicrobials as well as every five days intervals to serve as a prompt for prescribers to review the antimicrobial treatment course.

### Education and training

Misconceptions towards antimicrobial use remained one of the major limiting factor which contributed to antimicrobial misuse.^11^ Continuous medical education sessions highlighting topics in antimicrobial resistance trend and areas for practice improvements were conducted for hospital staffs as a supplementary AMS intervention to increase the awareness on prudent antimicrobial use. On the other hand, AMS core team members were provided with more advanced training through attachment to AMS rounds with ID physician in other teaching institutions. This was carried out to ensure good antimicrobial clinical practices were adopted and with the aim to promote stringent ASP implementation within hospital level.

### Program Evaluation

In this study, structured data collection sheet was utilized. The number of interventions recommended during AMS rounds and the percentage of agreement with AMS interventions by the treating physicians were tabulated and measured as the primary endpoints for process metrics. In addition, adherence to authorization policy upon initiation of restricted antimicrobials before and after AMS implementation were assessed as secondary process metric. The association of secondary endpoint between pre-intervention versus post-intervention was determined using Chi-square analysis. The level of significance in this study was expressed by p-value of less than 0.05. This study was approved by the Medical Research Ethical Committee, Ministry of Health Malaysia (ID NMRR-15-1875-28410).

## RESULTS

**Fig.2:**
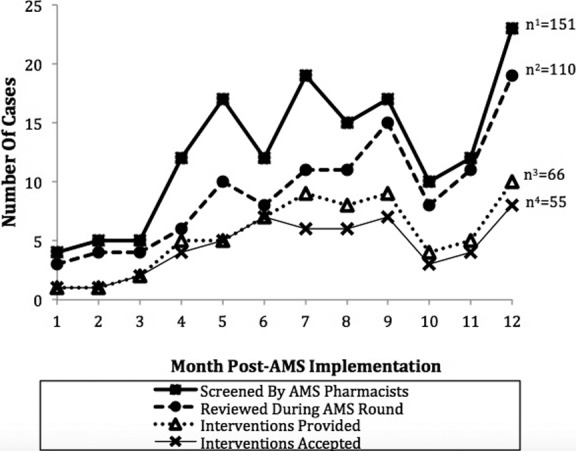
AMS process measures (cases screened, reviewed, intervened and accepted for intervention) throughout one year post-implementation.

**Fig.3:**
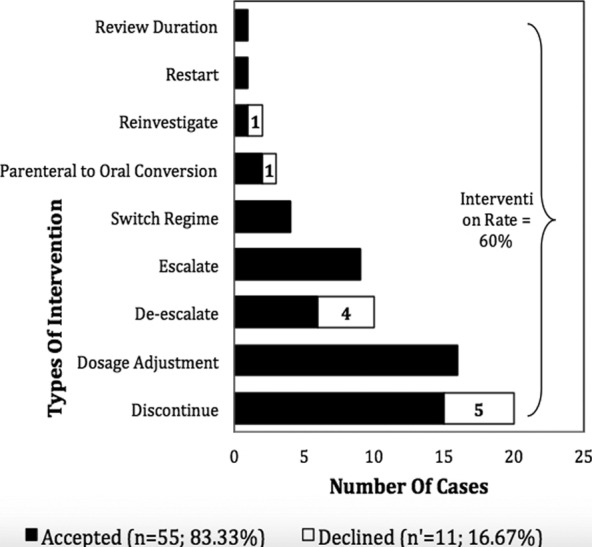
Types of AMS intervention (N=66).

Overall, the mean age of patients recruited under AMS review was 48.32 (± 16.532) years with a majority of them were males (65 patients; 59.1%) and Malays (74 patients; 67.3%). As depicted in Fig.2, the numbers of patients screened and reviewed increased throughout the year as the program evolved. Out of 110 patients reviewed during AMS rounds, recommendations were provided for 66 patients (60%) with the average acceptance rate of 83.33% (Fig.3). Majority of the interventions were to stop the antimicrobial therapy (30.3%) and the most common audited antimicrobials during AMS rounds was Piperacillin/Tazobactam (25.5%), followed by Meropenem (11.82%), Amoxicillin/Clavulanate and Vancomycin (8.18% respectively).

Throughout one year before ASP implementation, a total number of 310 restricted antimicrobial cases were initiated. Of note, only 188 (60.6%) cases were found to comply with restricted antimicrobial authorization policy. With the implementation of ASP in year 2015 and as the program evolved throughout the year, the restrictive policy had been expanded to include more types of antimicrobials (718 cases). The concordance rate towards authorization policy had been observed to increase steadily (71.59%; 514 cases). In order to facilitate the comparison between pre versus post ASP implementation, 9 selected antimicrobials had been selected for bivariate analysis (Table-I). Restrictive enforcement under ASP had been shown to improve significantly adherence rate towards antimicrobials authorization policy (p-value=0.004).

**Table-I:** Adherence towards restricted antimicrobial authorization policy before and after AMS implementation (based on 9 selected antimicrobials).

Adherence Towards Restricted Antimicrobials* Authorization Policy	Restricted Antimicrobial Cases (N= 739)		Analysis χ^2^; p-value

	Pre-AMS Implementation (n^a^=253)	Post-AMS Implementation (n^b^= 486)	
Yes	166	367	8.114; 0.004
No	87	119	

* Based on 9 selected antimicrobials (Cefepime, Cefoperazole/Sulbactam, Clindamycin, Ertapenem, Imipenem/Cilastatin, Meropenem, Piperacillin/Tazobactam, Polymyxin E and Vancomycin).

## DISCUSSION

This study describes the development and implementation of an ASP in a district hospital operating 268 beds. The successful implementation of this program marked the importance of judicious use of antimicrobials in combating the surge of antimicrobial resistance. Collaboration among the hospital administration, doctors, pharmacists, and supporting staffs determined the success in implementing this program.

Our study highlighted several challenges while implementing this program. Lack of funding and personnel were considered to be the primary barriers to ASP implementation in a survey of the IDSA Emerging Infections Network on programmatic strategies and barriers for ASP implementation.^12^ In our study, we were unable to allocate a dedicated full time pharmacist with specialized ID training due to inadequate personnel. The pharmacists involved only managed to rotate among themselves to handle ASP activities in addition to their daily routine works. Ideally, an ID physician should lead the AMS team. Unfortunately, routine visit from the ID physician of a tertiary center was not feasible. Thus, a medical specialist was assigned to lead the AMS team.

Additional challenges in the implementation of our program included organizing continuous medical education (CME) as well as data collection and management. CMEs were organized to promote awareness among the prescribers regarding judicious use of antimicrobials, and they were reminded on the authorization policies in starting restricted antimicrobials. Our study showed close collaboration under ASP stimulated change of an environment with positive reinforcement that enormously enhanced the adherence rate towards authorization policy prior to commencement of restricted antimicrobials.

In implementing the ASP, we observed the AMS team aspired to affect positive changes in antimicrobial prescribing behavior, pharmacists’ knowledge of stewardship principles, and clinicians’ confidence in AMS recommendations. This was highlighted in previous studies on significance and impact of behavioral change strategies in influencing antimicrobial prescribing from ASP.^13^ As the program evolved, we observed increasing referral from clinician for AMS team to review patient cases involving antimicrobial use and participation of prescribers during the AMS rounds to discuss specific cases. Furthermore, series of ASP education and training sessions also equipped our pharmacists to be more confident and competent to provide evidence-based interventions to the prescribing clinicians. This could be possibly evidenced by the wider gaps over the time between the number of cases of pharmacists screened and the number of cases that needed to be referred for AMS round for review. On the other hand, we also observed an increasing gap between cases reviewed under AMS round with those intervened under AMS round. This could be indicative of the routine antimicrobial prescribing practiced by various disciplines had been influenced and became lesser variations from those which were deemed appropriate by our AMS team as the time evolved. Proactive inter-disciplinary information sharing during AMS round had provided an opportunity to bring attention to the prescribers on inter-disciplines differences on antimicrobial prescribing practices, latest evidence-based recommendations, updates on antibiogram trending and ASP policies which could promoted prudent antimicrobial prescribing over the time.

The number of cases in which AMS recommendations accepted by the treating clinicians were encouraging. The acceptance rate in our study was reported to be higher compared with previous studies.^4^,^7^,^10^,^14^ Constructive support from the microbiology department played an important role in the success of the AMS program.^4^ Clinical microbiologist was part of the AMS team members, who provided useful information pertaining to patients’ cultures. This helped to streamline the antimicrobial use with narrower spectra and targeted to the causative organisms. On the other hand, our study revealed a minority of cases intervened were rejected by treating physicians, of which, it involved mostly suggestions to discontinue antimicrobials, followed by de-escalation and parenteral antimicrobial to oral conversion. The possible reasons are deviation from the attending physician’s clinical judgment, or it could be attributed by physician’s personal preferences.

Acknowledging the aforementioned challenges in implementing an ASP in a district hospital, collaboration among the hospital administrators was vital to ensure it a success. In view of our study was a first year report on implementation of ASP, only process metrics had been designed to be evaluated in this study. It was important to note that early program reports should focus on process measurements as clinical and microbiological measures may require a very long time horizon to show noticeable changes from the baseline.^15^ As advocated by Davey et al., sustained effect of ASP on clinical and microbiology outcomes should ideally be evaluated after one to two years or longer. 16 Hence, new measures will need to be planned for program improvement as it progresses. This will improve the patient’s care and avoid unnecessary or improper antimicrobial use. Specific clinical outcomes will need to be evaluated as the program matures include the hospital length of stay, morbidity, and mortality. Other aspects, include cost-effectiveness and impact on resistance rate, will be added on in the future.

## CONCLUSION

An ASP was successfully implemented in a district hospital. Despite various obstacles and challenges, they were overcome with collaboration from various parties. For ASP to be successful in a district hospital, addressing the specific needs of an individual institutions and for it to be set up on available resources, the limitations and advantages of each institution is vital. Moreover, recognizing and effective cope up with the communication barriers particularly among antagonizing physicians to ASP is important to ascribe changes towards stringent ASP practices. Future studies on its clinical outcomes are important to evaluate its effectiveness as well as focus on the improvement to the pre-existing strategies and measures.
